# Artemisinin-based antimalarial research: application of biotechnology to the production of artemisinin, its mode of action, and the mechanism of resistance of *Plasmodium* parasites

**DOI:** 10.1007/s11418-016-1008-y

**Published:** 2016-06-01

**Authors:** Paskorn Muangphrom, Hikaru Seki, Ery Odette Fukushima, Toshiya Muranaka

**Affiliations:** Department of Biotechnology, Graduate School of Engineering, Osaka University, 2-1 Yamadaoka, Suita, Osaka 565-0871 Japan; Continuing Professional Development Center, Department of Biotechnology, Graduate School of Engineering, Osaka University, 2-1 Yamadaoka, Suita, Osaka 565-0871 Japan

**Keywords:** *Artemisia annua*, Artemisinin, Resistant parasites, Malaria, Metabolic engineering, Mode of action

## Abstract

Malaria is a worldwide disease caused by *Plasmodium* parasites. A sesquiterpene endoperoxide artemisinin isolated from *Artemisia annua* was discovered and has been accepted for its use in artemisinin-based combinatorial therapies, as the most effective current antimalarial treatment. However, the quantity of this compound produced from the *A. annua* plant is very low, and the availability of artemisinin is insufficient to treat all infected patients. In addition, the emergence of artemisinin-resistant *Plasmodium* has been reported recently. Several techniques have been applied to enhance artemisinin availability, and studies related to its mode of action and the mechanism of resistance of malaria-causing parasites are ongoing. In this review, we summarize the application of modern technologies to improve the production of artemisinin, including our ongoing research on artemisinin biosynthetic genes in other *Artemisia* species. The current understanding of the mode of action of artemisinin as well as the mechanism of resistance against this compound in *Plasmodium* parasites is also presented. Finally, the current situation of malaria infection and the future direction of antimalarial drug development are discussed.

## Introduction

As a worldwide disease, malaria has been one of the main cause of illness and death in humans for over a century, especially in sub-Saharan Africa and Southeast Asia. More than 200 million cases of malaria are reported every year; in 2015, there were 214 million cases and 438,000 related deaths [1]. This disease is caused by five species of *Plasmodium* parasites: *P. falciparum*, *P. vivax*, *P. malariae*, *P. ovale*, and *P. knowlesi*. Among these, *P. falciparum* is the major cause of malaria infection in Africa, and *P. vivax* is the most widely distributed malaria-causing parasite globally [1]. Several antimalarial drugs have been developed since the seventeenth century. However, malaria-causing parasites have developed resistance to these conventional drugs, leading to treatment failure.

In response to the urgent need for new antimalarial drugs, Chinese scientists Professor Youyou Tu and her research group discovered artemisinin, the most effective antimalarial drug derived from *Artemisia annua* in 1971 [2]. Artemisinin is a sesquiterpene lactone with an endoperoxide bridge, which is necessary for antimalarial activity during multiple stages of parasite development [3–7]. Owing to its rapid action and high effectiveness against malaria, the combination of artemisinin derivatives and other antimalarial drugs, so-called artemisinin-based combination therapies (ACTs), has been recommended as the first-line treatments against malaria since 2006 [8]. ACTs have become the most powerful strategy to prevent malaria and related deaths. Professor Tu was then awarded the Nobel Prize in Physiology or Medicine in 2015 for the discovery of this effective antimalarial compound.

The demand for ACTs increases dramatically each year; yet, the production yield of artemisinin from *A. annua* is very low and varies widely from 0.01 to 2 % dry weight [9]. Alternative approaches, including plant breeding technologies, synthetic biology, and total and semi-syntheses of artemisinin, have been investigated to enhance the production and reduce the cost of this compound. In addition, the recent emergence of artemisinin-resistant *Plasmodium* parasites has also become a new challenge to scientists in the elucidation of the mechanism of resistance and identification of the new strategies for malaria treatment.

In this review, we summarize recent studies on the enhancement of artemisinin production and on artemisinin biosynthetic genes in other *Artemisia* species, conducted in our laboratory. In addition, the current understanding of the mode of action of artemisinin against malaria-causing parasites and, in turn, the mechanism of resistance of the parasites to this compound are also presented. Finally, the current situation of malaria infection and future directions, including ongoing studies on antimalarial drug development, are discussed.

## Discovery of artemisinin

Before the discovery of artemisinin, powder derived from cinchona tree bark had been used to treat malaria since the seventeenth century. The active compound from this plant, quinine, was first isolated in 1820 and was used as the only effective antimalarial compound until the 1920s. The quinine derivative chloroquine was developed as a new effective antimalarial drug once quinine-resistant *Plasmodium* strains appeared. During that time, the insecticide DDT was widely used to control the spread of infected mosquitoes as well. However, in the 1960s, increasing of chloroquine-resistant *Plasmodium* strains and DDT-resistant mosquitoes became a critical sign of the failure of malaria prevention and treatment [10].

In response to the urgent need for new antimalarial drugs, the Chinese government launched a national project against malaria called Project 523 in 1967 [2]. The group, led by Professor Youyou Tu, investigated more than 2000 Chinese herbs used as traditional Chinese medicines to treat fever. Among these herbs, an extract from *A. annua* showed highly effective inhibition against growth of malaria-causing parasites. The active antimalarial components were then extracted from the leaves of mature plants in 1971 [2, 10–12]. After purification, the active antimalarial compound, named qinghaosu or artemisinin, was obtained as colorless needle-like crystals. Its stereochemistry and chemical and X-ray crystal structures were determined and reported several years later [2, 10, 11, 13]. Clinical trials involving either a non-toxic *A. annua* extract or pure artemisinin have been conducted since 1972 by several groups, and all patients in these trials quickly recovered from the disease [11, 12]. These results clearly indicated that artemisinin is an effective antimalarial compound with rapid action and low toxicity.

Despite showing effective antimalarial activity, the low solubility of artemisinin in both oil and water becomes a therapeutic limitation of this compound. To address this problem, many scientists have developed semi-synthetic drugs and synthesized artemisinin derivatives with higher solubility. Some of these artemisinin derivatives, which have been used until the present, include dihydroartemisinin, artemether, and artesunate [14]. In addition, the combination of artemisinin or its derivatives with other conventional antimalarial drugs greatly increased the parasite clearance rate in patients and was first recommended as a new strategy for malaria treatment in 1984 [15]. This strategy, known as ACT, has been recommended by the World Health Organization (WHO) as a first-line treatment for malaria to prevent recurrence and development of resistance in malaria-causing parasites, whereas the monotherapy is considered as an inappropriate treatment [2, 8, 13, 14, 16].

## Biosynthesis of artemisinin and expression pattern of artemisinin biosynthetic genes in *A. annua*

**Fig. 1:**
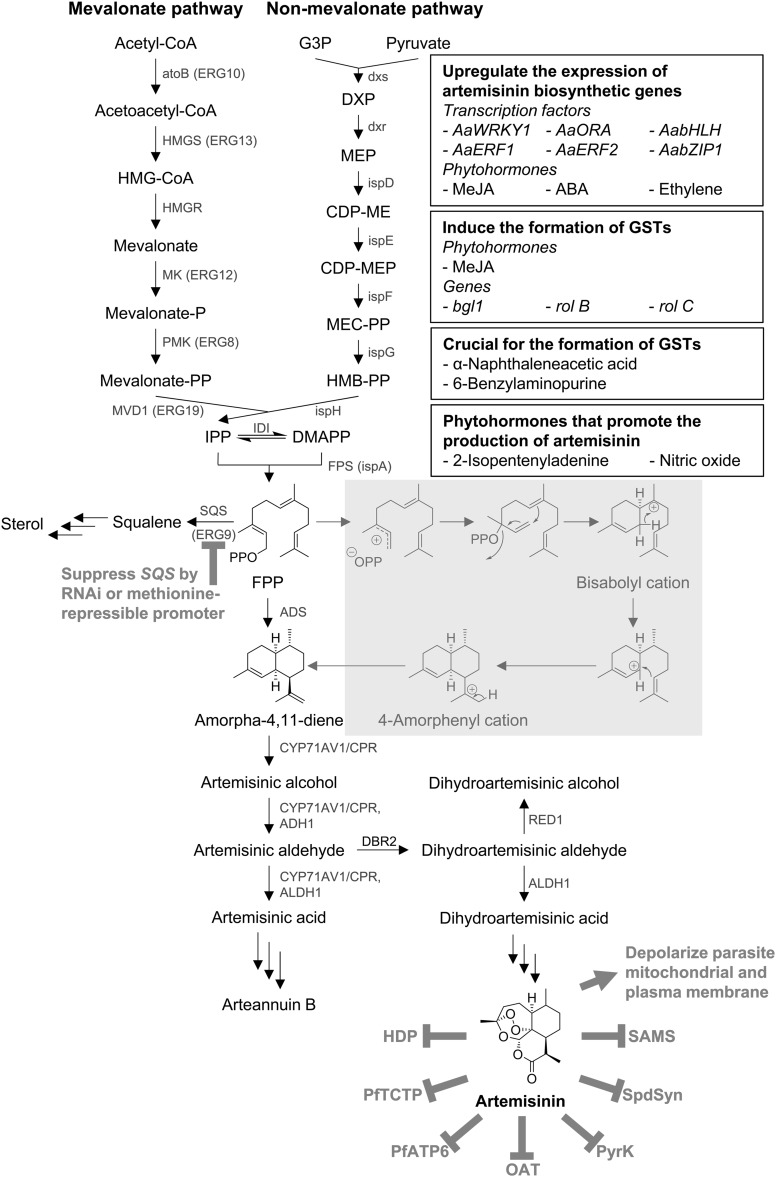
Summary of artemisinin biosynthesis, transgenic approaches to enhance artemisinin production, and artemisinin mode of action. The enzymes responsible for each reaction are indicated next to the *arrows*. Suppression of competing pathways and artemisinin activity and its targets are shown in *bold*. Transgenic approaches regulating artemisinin production are shown in black boxes. Cyclization mechanism of FPP to generate amorpha-4,11-diene is highlighted in *gray*. Full names of intermediates and enzymes involved in the pathway are as follows: *HMG-CoA* 3-hydroxy-3-methylglutaryl-coenzyme A, *G3P* glycerol-3-phosphate, *DXP* 1-deoxy-d-xylulose 5-phosphate, *MEP* 2*C*-methyl-d-erythritol 4-phosphate, *CDP-ME* 4-diphosphocytidyl-2*C*-methyl d-erythritol, *CDP-MEP* CDP-ME 2-phosphate, *MEC-PP* 2*C*-methyl-d-erythritol 2,4-cyclodiphosphate, *HMB-PP* (*E*)-4-hydroxy-3-methyl-but-2-enyl pyrophosphate, *IPP* isopentenyl pyrophosphate, *DMAPP* dimethylallyl pyrophosphate, *atoB* (*ERG10*) acetoacetyl-CoA thiolase, *HMGS* (*ERG13*) HMG-CoA synthase, *HMGR* HMG-CoA reductase, *MK* (*EGR12*) mevalonate kinase, *PMK* (*ERG8*) phosphomevalonate kinase, *MVD1* (*ERG19*) mevalonate pyrophosphate decarboxylase, *dxs* DXP synthase, *dxr* DXP reductase, *ispD* CDP-ME synthase, *ispE* CDP-ME kinase, *ispF* MEC-PP synthase, *ispG* HMB-PP synthase, *ispH* HMB-PP reductase, *IDI* IPP isomerase, *FPS* (*ispA*) farnesyl pyrophosphate (FPP) synthase, *SQS* (*ERG9*) squalene synthase, *ADS* amorpha-4,11-diene synthase, *CYP71AV1* amorpha-4,11-diene 12-monooxygenase, *CPR* cytochrome P450 reductase, *ADH1* alcohol dehydrogenase 1, *ALDH1* aldehyde dehydrogenase 1, *DBR2* artemisinic aldehyde Δ11(13) reductase, *RED1* dihydroartemisinic aldehyde reductase 1 (color figure online)

A precursor of artemisinin, farnesyl pyrophosphate (FPP, C_15_), is synthesized from two C-5 isoprenoid units derived from the cytosolic mevalonate (MVA) pathway and one isoprenoid unit derived from the non-mevalonate (MEP or DXP) pathway [17, 18]. FPP is cyclized to amorpha-4,11-diene by amorpha-4,11-diene synthase (ADS) [19–21] via the generation of bisabolyl and 4-amorphenyl cation intermediates [22, 23] (Fig. 1). The following step is the oxidation of amorpha-4,11-diene to artemisinic alcohol by amorpha-4,11-diene 12-monooxygenase (CYP71AV1) [24]. This enzyme also catalyzes the oxidation of artemisinic alcohol to artemisinic aldehyde and artemisinic acid. In addition, alcohol dehydrogenase 1 (ADH1) and aldehyde dehydrogenase 1 (ALDH1) also show specific oxidation activity on artemisinic alcohol into artemisinic aldehyde and on artemisinic aldehyde into artemisinic acid, respectively [25, 26]. Artemisinic acid was thought to be the last precursor of artemisinin. However, it has been revealed that this compound is converted non-enzymatically into arteannuin B and related compounds, rather than artemisinin [27]. The next step of artemisinin biosynthesis is the reduction of artemisinic aldehyde into dihydroartemisinic aldehyde by artemisinic aldehyde Δ11(13) reductase (DBR2) [28]. Then, ALDH1 oxidizes dihydroartemisinic aldehyde into dihydroartemisinic acid, which is converted non-enzymatically into artemisinin [26, 29], as shown in Fig. 1. Rydén et al. [30] discovered dihydroartemisinic aldehyde reductase 1 (RED1), which reduces dihydroartemisinic aldehyde into dihydroartemisinic alcohol. Although the role of RED1 in artemisinin biosynthesis is still unclear, it has been suggested that the silencing of *RED1* might increase the production of artemisinin in *A. annua*.

Artemisinin is produced mainly in glandular secretory trichomes (GSTs) and its accumulation level declines as plants mature. Olofsson et al. [31] showed that GSTs of *A. annua* are found in all aerial tissues of plants, but not in roots or hairy roots. The density of GSTs is highest in flower buds and young leaves and decreases as leaves age.

The expression pattern of genes involved in the artemisinin biosynthetic pathway has been investigated extensively for over a decade. The expression of genes in the upstream pathway shows no correlation with the density of GSTs or the accumulation levels of artemisinin intermediates [32]. In contrast, the expression of genes in the downstream pathway is consistent with the density of GSTs in each tissue. The expression of *ADS* is highest in GSTs, high in flower buds and young leaves, low in stems, negligible in old leaves and hairy roots, and not detected in roots [31, 33–37]. *CYP71AV1*, *DBR2*, and *ALDH1* showed similar expression patterns: highest in GSTs and very low in stems and roots. In hairy roots, the expression levels of *CYP71AV1* and *DBR2* are relatively low, but the expression of *ALDH1* is negligible [24, 26, 28, 31]. The expression levels of *CYP71AV1* and *DBR2* in leaves and flowers show similar patterns, as they are high in leaf primordia and flower buds but decrease as leaves and flowers develop [38–40]. The expression pattern of *ALDH1* in leaves at different stages is similar to those of *CYP71AV1* and *DBR2* [31]. Although there is no report on the expression level of *ALDH1* during different stages of flowering, this gene shows higher expression in flowers than in leaves [26, 31]. The expression of *RED1* is relatively low in flower buds, young leaves, and stems. In contrast, the expression of this gene is much higher in old leaves and roots than in young leaves [30, 31]. Interestingly, the expression of *RED1* is approximately 50-fold higher in hairy roots compared with old leaves. Nevertheless, the function of RED1 in hairy roots has not been established [31].

The expression levels of *ADS* and *ALDH1*, as well as their enzymatic activities in high-artemisinin-producing and low-artemisinin-producing *A. annua* cultivars, show no differences. Even though the expression levels of *CYP71AV1* in these two cultivars are similar, CYP71AV1 in a high-artemisinin-producing cultivar shows lower enzyme activity, which is suitable for the change in metabolic flux to dihydro-analogues and artemisinin production [41]. In contrast, the activity of DBR2 in both cultivars shows no significant difference, but the gene encoding this enzyme shows considerably higher expression levels in high-artemisinin-producing cultivars than in low-artemisinin-producing cultivars [42].

## Mode of action of artemisinin

Before artemisinin can exert its action, the endoperoxide bridge has to be activated to generate the free radical species. Two activation pathways of artemisinin have been suggested, namely the mitochondrial and heme-mediated degradation pathways [43]. Mitochondria-activated artemisinin is involved in lipid peroxidation inducing cytotoxicity via the generation of reactive oxygen species (ROS) and depolarization of the parasite mitochondrial and plasma membranes [43–47]. In the heme-mediated pathway, two activation models (i.e., a reductive scission model and an open peroxide model) have been proposed, both of which lead to the generation of an active carbon-centered radical [48]. Even though the non-heme Fe^2+^ ion was suggested to bind and activate artemisinin [7], recent studies showed that heme plays a predominant role in artemisinin activation rather than the Fe^2+^ ion [5]. In *Plasmodium* spp., heme is produced via endogenous heme biosynthesis at the early ring stage and via hemoglobin digestion at the trophozoite stage. However, the level of heme biosynthesized endogenously in the parasites is much lower than its production via hemoglobin digestion, suggesting that hemoglobin-derived heme plays a major role in artemisinin activation [5, 49]. Recently, Xie et al. [50] reported that falcipains FP2a and FP3 (two main cysteine protease hemoglobinases) are also involved in the potential activation of artemisinin at an early ring stage.

After hemoglobin digestion, the heme detoxification protein (HDP) can trigger the conversion of free heme to hemozoin, which is essential for parasite survival [4, 51]. However, the formation of the artemisinin-free heme complex shows an inhibitory effect on this conversion [51]. A translationally controlled tumor protein (PfTCTP) was also reported as a potential target of artemisinin, as it could form a covalent bond with this protein, resulting in protein malfunction [52, 53]. Eckstein-Ludwig et al. [54] showed that artemisinin specifically mediated the inhibition of PfATP6, an orthologous sarco/endoplasmic reticulum Ca^2+^-ATPase (SERCA), outside the food vacuole. Recently, five enzymes involved in the key metabolic pathways of the parasite were also reported as potential targets of artemisinin, namely ornithine aminotransferase (OAT), pyruvate kinase (PyrK), l-lactate dehydrogenase (LDH), spermidine synthase (SpdSyn), and *S*-adenosylmethionine synthetase (SAMS). All of them are covalently modified by the interaction with artemisinin, resulting in the irreversible malfunction of enzyme activities [5].

## Enhancement of artemisinin production

The demand for artemisinin increases every year. Even though total synthesis of artemisinin from commercially available chemicals or semi-synthesis from its intermediates have been reported, all of those methods are costly and require several synthesis steps [55, 56]. In this review, we summarize recent studies regarding four approaches to enhance the production of artemisinin: (1) plant breeding technologies, (2) overexpression of genes involved in the artemisinin biosynthetic pathway, (3) direct or indirect upregulation of artemisinin biosynthesis, and (4) heterologous production.

### Plant breeding technologies

Conventional plant breeding techniques to select high-artemisinin-producing cultivars have been used for decades. These techniques include cultivation of *A. annua* and collection of cultivars with the desired properties. At present, a robust hybrid *A. annua* is generated from the combination of high-artemisinin-producing and vigorous cultivars to increase the production yield of artemisinin to more than 2 % dry weight [57–59]. Recently, an alternative approach to increase the production of artemisinin from the cultivation of high-artemisinic acid or dihydroartemisinic acid-producing cultivars was proposed, since a method for the semi-synthesis of artemisinin from these two precursors has been developed [56, 60].

Scientists at the University of York used advanced breeding techniques to evaluate the distribution of traits that contribute to artemisinin yield [61]. From the screening of 23,000 strains, they succeeded in identifying genes and molecular markers for fast-track breeding, enabling the construction of a detailed genetic map of *A. annua* with nine linkage groups. The established quantitative trait loci (QTL) map is also applicable for rapid identification of *A. annua* parental lines with useful traits for plant breeding. Two hybrids, called Hybrid 1209r *Shennong* and Hybrid 8001r *Zenith*, were developed with high artemisinin productivity of up to 36.3 and 54.5 kg/ha, respectively. The diallel cross approach to determine the combining ability of the robust parental lines for the production of artemisinin high-yielding *A. annua* hybrids was also developed by the same group and showed consistent results with the QTL-based molecular breeding approach [62].

Hairy root culture is another method to enhance the production of secondary (specialized) metabolites, owing to its rapid growth capabilities [63]. Transformation protocols to obtain hairy roots containing artemisinin from this plant have been reported [64, 65]. In our laboratory, we also attempted to establish the conditions for *A. annua* hairy root cultivation. However, we still could not detect even trace amounts of artemisinin or its intermediates from the extract of hairy root cultures by GC–MS (unpublished data). Artemisinin biosynthetic genes are highly expressed in trichomes but almost negligible in root tissue [31, 33–40], suggesting that the production of this compound by hairy root cultures could be somewhat difficult. Therefore, the most suitable conditions for hairy root cultures to enhance production of artemisinin must be investigated. In addition, the identification of artemisinin production from root extracts requires extreme care, and NMR spectroscopic and mass spectrometric analyses are required.

### Overexpression of genes involved in artemisinin biosynthetic pathway

Metabolic engineering of *A. annua* by overexpressing genes involved in artemisinin biosynthesis has been given more attention during the last 20 years. To obtain successful transformants, several parameters for *Agrobacterium tumefaciens*-mediated transformation, such as the concentration of antibiotics, method and duration of co-cultivation, and phytohormones supplied for plant regeneration, have been optimized [66–71]. Among various explants available for transformation, stem internodes and young inflorescence seem to be the most appropriate [70–72]. Phytohormones α-naphthaleneacetic acid and 6-benzylaminopurine are crucial for GST development in young leaves, and root generation also affects GST size [73]. Recently, Kiani et al. [72] developed miniprep methods using *A. tumefaciens*- and *Agrobacterium rhizogenes*-mediated transformation. This method exhibits higher transformation rates with faster development of transformants within 3–4 weeks compared with other methods.

The overexpression of several genes involved in artemisinin biosynthesis in *A. annua* has been evaluated. Overexpression of farnesyl pyrophosphate synthase (*FPS*) increased artemisinin production up to 2- to 3.6-fold higher than that in the control [74, 75]. Overexpressing *CYP71AV1* and its redox partner cytochrome P450 reductase (*CPR*) in artemisinin biosynthesis could increase artemisinin content *in planta* by 38 % [76]. Xiang et al. [77] generated *dxr*- and *CYP71AV1*/*CPR*-overexpressing *A. annua* and found that both transformants increased the production of artemisinin. The overexpression of *DBR2* increased the production of artemisinin as well as its precursor dihydroartemisinic acid, up to twofold, compared with non-transgenic plants. It also increased production of artemisinic acid up to 5.48- to 9.06-fold and arteannuin B up to twofold [78]. The reason why overexpression of *DBR2* enhanced biosynthesis of artemisinic acid and arteannuin B has not been revealed. However, Yuan et al. [78] hypothesized that excess dihydroartemisinic acid might be converted into artemisinic acid *in planta*.

Overexpression of multiple genes involved in artemisinin biosynthesis could greatly increase the production of artemisinin *in planta*. Chen et al. [79] showed that the co-overexpression of *FPS*, *CYP71AV1*, and *CPR* increased artemisinin levels in *A. annua* up to 3.6-fold. The co-overexpression of *HMGR* and *FPS* increased production of artemisinin up to 1.8-fold higher than that in the control [80]. Alam et al. [81, 82] co-overexpressed *HMGR* and *ADS* in *A. annua* and found greatly increased artemisinin levels, up to 7.65-fold, in this transgenic line.

Suppressing the expression of genes involved in the pathways competing with artemisinin biosynthesis is another approach to enhance artemisinin content *in planta*. Zhang et al. [83] used RNAi techniques to suppress the expression of *SQS*, the first committed gene in sterol biosynthesis. The suppression of this gene enhanced the production of artemisinin up to 3.14-fold.

### Direct or indirect upregulation of artemisinin biosynthesis

The effect of several stresses on production of artemisinin in *A. annua* has been analyzed since the 1990s. These stresses usually lead to the generation of ROS (required for the last non-enzymatic step in artemisinin biosynthesis) or upregulate the expression of artemisinin biosynthetic genes [84–87]. Details of the stresses placed on artemisinin production have been summarized previously [88, 89], and the appropriate cultivation conditions of *A. annua* were suggested [9].

Some transcription factors upregulated the expression of artemisinin biosynthetic genes and promoted production of artemisinin in *A. annua*. The WRKY1 transcription factor is thought to bind to the W-box *cis*-acting elements of promoters to promote gene expression. It is also involved in the regulation of plant defense responses and developmental and physiological processes. Ma et al. [33] showed that the transcript levels of *HMGR*, *ADS*, *CYP71AV1*, and *DBR2* were induced in transient *AaWRKY1*-overexpressing leaves. Furthermore, the specific overexpression of this transcription factor in GSTs increased transcript levels of *CYP71AV1* up to 33-fold, compared with the wild type [90]. *AaORA*, one of the APETALA2/ethylene response factor (AP2/ERF) transcription factor involved in plant responses to biotic and abiotic stresses, showed a similar expression pattern to those of *ADS*, *CYP71AV1*, and *DBR2*. The overexpression of this transcription factor led to the upregulation of the expression levels of *ADS*, *CYP71AV1*, and *DBR2in planta* and promoted artemisinin production [91]. Yu et al. [92] also reported the enhancement of artemisinin production via overexpression of two transcription factors from the same family, *AaERF1* and *AaERF2*, which bind to the promoter regions of *ADS* and *CYP71AV1*. Another transcription factor that positively regulates the biosynthesis of artemisinin is a basic helix-loop-helix (bHLH) transcription factor, involved in metabolic regulation of various hormones, developmental processes, and regulation of light signaling, iron and phosphate homeostasis, and various abiotic stresses [93]. Recently, Zhang et al. [94] reported that a basic leucine zipper transcription factor (AabZIP1) binds to the ABA-responsive elements (ABRE) of *ADS* and *CYP71AV1* promoters and upregulates the expression of *ADS*, *CYP71AV1*, *DBR2*, and *ALDH1*.

Several phytohormones upregulating artemisinin biosynthesis have been reported. Treatment with salicylic acid upregulates the expression of *HMGR* and *ADS*, as well as induces ROS generation, driving the conversion of dihydroartemisinic acid into artemisinin [95]. Methyl jasmonate (MeJA) promotes the formation of GSTs and enhances the expression of several genes involved in the artemisinin biosynthetic pathway and related transcription factors (*ORA* and *ERF1*), leading to the enhancement of artemisinin production [96–98]. This phytohormone also regulates trichome-specific fatty acyl-CoA reductase 1 (*TFAR1*), ABCG transporter unigenes (*AaABCG6* and *AaABCG7*), and allene oxide cyclase (*AaAOC*) [96, 99, 100]. TFAR1 is involved in the formation of cuticular wax during GST expansion in *A. annua*. *AaABCG6* and *AaABCG7* are ATP-binding cassette transporter G, involved in the development of trichome cuticle and may share a common regulatory system with *ADS* and *CYP71AV1*. *AaAOC* is involved in JA biosynthesis. The expression of this gene may be upregulated by treatment with not only MeJA but also ABA and ethylene [100]. The overexpression of the ABA receptor, *AaPYL9*, also improves the sensitivity of ABA and promotes artemisinin biosynthesis after ABA treatment [99, 101].

The enhancement of artemisinin production can be achieved by increased GST density. Singh et al. [102] reported that the expression of *bgl1*, encoding β-glucosidase from *Trichoderma reesei*, in *A. annua* improved the density of GSTs in flowers up to 66 % and increased the production of artemisinin up to five-fold compared with the control. The expression of *rolB* and *rolC* of *A. rhizogenes* also increases GST density and upregulates the expression of *ADS*, *CYP71AV1*, *ALDH1*, and *TFAR1*. Artemisinin content is then increased 2- to 9-fold and 4-fold in *rolB*- and *rolC*-expressing plants, respectively [103].

Co-cultivation of an endophytic fungus *Piriformospora indica* and a nitrogen-fixing bacterium *Azotobacter chroococcum* with *A. annua* increases artemisinin content up to 70 % [104]. This dual symbiosis also shows a positive effect on plant height, dry weight, and leaf yield. Another example of using symbiosis to increase the production of artemisinin was reported using *Glomus mosseae* and *Bacillus subtilis* [105]. Although clear evidence for the effect of this symbiosis on the enhancement of artemisinin production is still unknown, Arora et al. [104] suggested that it might be due to improved growth and nutrient status of the plant.

### Heterologous production

Metabolic engineering of several platforms, such as *Nicotiana benthamiana* or chloroplasts, has been conducted. Although *ADS* and *CYP71AV1* were introduced into *N. benthamiana*, the production of artemisinic acid 12-β-diglucoside, instead of artemisinic acid, was detected at 39.5 mg/kg fresh weight (FW) [106]. The production yield of artemisinic acid in tobacco chloroplasts was also very low (0.1 mg/g FW) [107].

The production of plant natural compounds in microorganisms is an alternative approach with several advantages. The metabolic pathways in microorganisms could be modified to produce various types of natural compounds, including isoprenoids, alkaloids, and phenylpropanoids. Microorganisms can grow rapidly, allowing shorter production time compared with the biosynthesis of desired natural compounds in plants. Scaling up production to industrial scale is also possible [108].

The production of artemisinin precursors in microorganisms was first reported in 2003. Martin et al. [109] expressed entire genes encoding the MVA pathway from yeast *Saccharomyces cerevisiae* in *Escherichia coli* to increase the intracellular concentration of FPP. To prevent the rapid loss of highly volatile amorpha-4,11-diene during culturing, the culture media was overlaid with dodecane to trap amorpha-4,11-diene, referred to as a two-phase partitioning bioreactor. As a result, they recovered the volatilized amorpha-4,11-diene, improving production titers from 24 mg/L to approximately 500 mg/L in a fed-batch bioreactor [110].

The coexpression of *MevT* operon with extra copies of *HMGR* reduced the accumulation of toxic HMG-CoA and increased production of mevalonate by threefold [111]. The replacement of *lac* by *lacUV5* promoter with a codon-optimized *MevT* and an additional copy of *MK* also led to the increase in artemisinin production [112]. Tsuruta et al. [113] succeeded in enhancing amorpha-4,11-diene production in *E. coli* up to 27.4 g/L by replacing yeast HMGS and HMGR with the equivalent enzymes from gram-positive bacteria *Staphylococcus aureus*.

Engineering of the MEP pathway and membrane efflux transporters to improve the production of amorpha-4,11-diene in *E. coli* has been reported as well [114–117]. However, there are many issues regarding the expression of membrane-bound cytochrome P450s in this bacterium posing a limitation on the production of the subsequent oxidized compounds. To overcome these problems, Chang et al. [118] engineered the N-terminal transmembrane domain of the codon-optimized *CYP71AV1* and coexpressed it with CPR from *A. annua*. As a result, production of artemisinic acid (105 mg/L) in this *E. coli* strain was obtained. Two years later, the same group replaced *CYP71AV1* by engineered P450 from gram-positive bacteria *Bacillus megaterium* (P450_BM3_) and could produce artemisinic-11*S*,12-epoxide at higher than 250 mg/L successfully [119]. From this finding, a novel semi-biosynthetic route for the production of artemisinin stemming from the cleavage of this epoxide followed by several oxidation steps was proposed.

Yeast is another attractive host for the production of artemisinin precursors as it produces FPP for sterol biosynthesis via the MVA pathway. Since the MVA pathway in *S. cerevisiae* has been characterized, *ADS* was introduced into this yeast, and an amorpha-4,11-diene-producing yeast strain was generated successfully [120]. While there are many issues concerning the expression of cytochrome P450s in *E. coli*, the expression of this gene in yeast is much more feasible. Therefore, *CYP71AV1* and *CPR* were coexpressed, and all genes involved in the MVA pathway were upregulated either directly or indirectly. The competing pathway (sterol biosynthetic pathway) was also downregulated using a methionine-repressible promoter to improve the production of artemisinic acid in the yeast expression system. As a result, this transgenic yeast strain produced artemisinic acid at up to 100 mg/L [121, 122]. Several factors were further optimized for the production of artemisinic acid in an industrial fermenter. For example, the carbon source for growing yeast in a fermenter was switched from glucose to galactose, and the oxygen transfer rate was controlled. With this development, called the galactose fed-batch process controlled by the DO-stat algorithm, the artemisinic acid titer increased to 2.5 g/L [123].

Despite conferring a higher production yield of artemisinic acid, the use of galactose is costly and not applicable, especially in developing countries. Thus, lower-cost chemicals are needed as carbon sources. Yeast with *GAL1*, *GAL7*, *GAL10*, and *GAL80* deletions was generated to exclude the use of galactose, and ethanol was alternatively used as a carbon source. Two additional copies of truncated *HMGR* (*tHMG1*) were integrated into this yeast strain. As a result, the production of amorpha-4,11-diene was increased up to more than 40 g/L [124]. Further development was performed by the introduction of artemisinin biosynthetic genes, *CYP71AV1*, *CPR*, *ADH1*, and *ALDH1*, to oxidize amorpha-4,11-diene into artemisinic acid. Cytochrome * b*_5_ (*CYB5*) was also introduced into this strain as it can accelerate cytochrome P450 reactions [125]. High-level production of artemisinic acid, at 25 g/L, was thereby achieved. The semi-synthesis of artemisinin from artemisinic acid was also optimized, and the overall yield after purification increased to 40–45 % [126, 127]. A potent coupled chromatography–crystallization method to purify artemisinin was then developed, and the recovery yield of this antimalarial compound from the reaction mixture increased to 61.5 %, with 99 % purity [128]. All of the transgenes and modifications to several heterologous hosts mentioned here are summarized in Table 1.

**Table 1:** Heterologous production of artemisinin intermediates

Host	No.	Transgenes or modifications	Product	Yield	References
*N. benthamiana*	1	*P*_*35S*_-*tHMGR*-*FPS*-*ADS*, *P*_*35S*_-*CYP71AV1*	Artemisinic acid 12-β-diglucoside	39.5 mg/kg FW	[106]
Tobacco chloroplasts	2	*P*_*rrn16S*_-*atoB*-*HMGS*-*HMGR*-*MK*-*PMK*-*MVD1*, *P*_*psbA*_-*E. coli* *IDI*-*FPS*-*ADS*-*CYP71AV1*-*AaCPR*	Artemisinic acid	0.1 mg/g FW	[107]
*E. coli*	3	*P*_*lac*_-*MevT* ^a^, *P*_*lac*_-*MBIS* ^b^, *P*_*trc*_-*ADS*	Amorpha-4,11-diene	24 mg/L	[109]
	4	Same as 3 but overlaid with dodecane	Amorpha-4,11-diene	500 mg/L	[110]
	5	*P*_*BAD*_-*MevT*, *P*_*BAD*_-*tHMGR1*	Mevalonate	Threefold from CT^c^	[111]
	6	*P*_*lacUV5*_-*MevT* (codon opt.)-*MBIS*, *P*_*trc*_-*ERG12* (codon opt.)-*ADS*	Amorpha-4,11-diene	293 mg/L	[112]
	7	*P*_*lacUV5*_-*MevT* (codon opt.) with *HMGS* and *HMGR* from *S. aureus*, *P*_*lac*_-*MBIS*, *P*_*lac*_-*ADS*	Amorpha-4,11-diene	27.4 g/L	[113]
	8	*P*_*BAD*_-*dxs*-*IDI*-*ispDF*, *ADS* with *Δpts* and optimized medium	Amorpha-4,11-diene	182 mg/L	[114]
	9	*P*_*TM2*_-*galP*-*glk*, *P*_*T7*_-*dxs*-*IDI*-*ispA*-*ADS*	Amorpha-4,11-diene	201.2 mg/L	[115]
	10	*AcrB*, *TolC* (*x2*), *ADS* (codon opt.)	Amorpha-4,11-diene	404.83 mg/L	[116]
	11	*P*_*BAD*_-*dxs*-*IDI*-*ispDF*, *P*_*araBAD*_-*ADS*, *P*_*TM1*_-*macAB*-*TolC*	Amorpha-4,11-diene	~30 mg/L/OD	[117]
	12	Same as 3 with *CYP71AV1* (codon opt., engineered N-terminal transmembrane)-*AaCPR*	Artemisinic acid	105 mg/L	[118]
	13	Same as 12 but replaced *CYP71AV1* with *P450*_*BM3*_	Artemisinic-11*S*,12-epoxide	250 mg/L	[119]
*S. cerevisiae*	14	*P*_*GAL1*_-*ADS*	Amorpha-4,11-diene	600 μg/L	[120]
	15	*P*_*GAL1*_-*tHMGR P*_*GAL1*_-*upc2*-*1 erg9::P*_*MET3*_-*ERG9 P*_*GAL1*_-*tHMGR P*_*GAL1*_-*ERG20*, *P*_*GAL1*_-*ADS P*_*GAL10*_-*CYP71AV1 P*_*GAL1*_-*AaCPR*	Artemisinic acid	100 mg/L	[121, 122]
	16	Same as 15 with optimized culture condition	Artemisinic acid	2.5 g/L	[123]
	17	*gal80Δ::nat* ^*r*^ *MAT a erg9Δ∷kan* ^*r*^ *P*_*MET3*_-*ERG9*, *leu2*-*3,112∷HIS P*_*GAL1*_-*MVD1 P*_*GAL10*_-*ERG8 his3Δ1∷HIS P*_*GAL1*_-*ERG12 P*_*GAL10*_-*ERG10ade1Δ∷P*_*GAL1*_-*tHMG1 P*_*GAL10*_-*IDI1 ADE1 ura3*-*52∷P*_*GAL1*_-*tHMG1 P*_*GAL10*_-*ERG13 URA3trp1*-*289∷P*_*GAL1*_-*tHMG1 P*_*GAL10*_-*ERG20 TRP1* [*pAM322*]	Amorpha-4,11-diene	41 g/L	[124]
	18	Same as 17. but replaced *gal80Δ::nat* ^*r*^ with *gal1Δ gal7Δ gal10Δ::hphA*	Amorpha-4,11-diene	37 g/L	[124]
	19	*gal1Δ,gal10Δ,gal7Δ::P*_*GAL3*_-*CPR1natA*, *erg9Δ::dsdAP*_*CTR3*_- *ERG9*, *leu2*-*3,112::kanAP*_*GAL7*_-*AaCYB5P*_*GAL1*_-*ERG19P*_*GAL10*_- *ERG8*, *ade1Δ::P*_*GAL1*_-*tHMG1P*_*GAL10*_-*IDI1_ADE1*, *his3Δ1::hphAP*_*GAL7*_-*AaALDH1P*_*GAL1*_-*ERG12P*_*GAL10*_-*ERG10*, *ura3*-*52:: P*_*GAL1*_-*tHMG1P*_*GAL10*_-*ERG13hisG*, *trp1*-*289:: P*_*GAL1*_- *tHMG1P*_*GAL10*_-*ERG20TRP1*, *ndt80Δ::P*_*TDH1*_-*HEM1HIS3PPGK1*-*CTT1*, *gal80Δ::URA3P*_*GAL7*_-*AaADH1*, [*pAM552: 2μ*-*LEU2d P*_*GAL1*_-*ADS P*_*GAL10*_-*CYP71AV1*]	Artemisinic acid	25 g/L	[126, 127]

^a^ *MevT* operon consists of *atoB*-*HMGS*-*tHMGR*

^b^ *MBIS* operon consists of*ERG12*-*ERG8*-*MVD1*-*IDI*-*ispA*

^c^Production yield as compared to control (CT)

## Artemisinin biosynthetic genes in non-artemisinin-producing *Artemisia* species

Some studies reported that artemisinin is produced in other *Artemisia* species [129–134]. However, we attempted to isolate artemisinin from other *Artemisia* species but failed to detect any trace amounts of artemisinin or its intermediates (unpublished data). Thus, we analyzed the expression of genes highly homologous to artemisinin biosynthetic genes in these species. Firstly, we selected *A. afra* and *A. absinthium* as they are widely cultivated in Africa and exhibit anti-plasmodial activity [135–138]. Putative *ADS* orthologs were not expressed in either *A. afra* or *A. absinthium* [139]. However, we detected the expression of putative *CYP71AV1* orthologs in both species. Functional analysis revealed that these orthologous enzymes show similar catalytic activities to their correspondent in *A. annua* on the oxidation of amorpha-4,11-diene into artemisinic acid [139]. We also detected the expression of *DBR2* ortholog in *A. absinthium*, and the encoded enzyme showed comparable activity to that of *A. annua* DBR2 [140]. In addition, we showed that this plant can convert the fed artemisinin intermediates into the following products along the biosynthetic pathway of artemisinin [140]. Our findings suggest that ADS might be a limiting factor for the production of artemisinin *in planta*, and *A. absinthium* could be an alternative host for artemisinin production. The introduction of *ADS* into *A. absinthium* might lead to the generation of artemisinin-producing *A. absinthium*, which could be used as an alternative approach to produce artemisinin in other *Artemisia* species. To prove this hypothesis, this research is now ongoing in our laboratory.

## Next challenge: artemisinin-resistant *Plasmodium* parasites

Artemisinin is the most effective antimalarial drug and has been used as an ACT to treat malaria for over a decade. However, the emergence of artemisinin-resistant *Plasmodium* parasites in Southeast Asia, prolonging the parasite clearance rate in patients, has been reported recently and has become a critical issue [141–144]. No correlation between resistance and other previously proposed candidate targets of artemisinin (*PfATP6* and *PfTCTP*) was detected [145]. However, it has been suggested that the resistance occurs predominantly during the early ring stage of parasite development as a result of the multiple forms of mutations in the *PF3D7_1343700* kelch propeller domain (K13-propeller) on chromosome 13 [146–155]. K13-propeller mutations lead to the increase of phosphatidylinositol-3-kinase (PfPI3K), which is required for the mediation of cell signaling and survival [156, 157], and prolong parasite development at the ring stage when the activation level of artemisinin is rather low [5, 7, 158]. The B subfamily of ABC transporters, known as multidrug resistance proteins (MDR), also promotes artemisinin resistance. In artemether–lumefantrine post-treatment infections, alleles of *Pfmdr1* tended to have 86N, 184F, and 1246D, rather than the common YYY haplotype, and increased the number of treatment failures [159]. The deletion of *Pfmdr5* induced greater sensitivity to artemisinin treatment, suggesting that this gene might contribute to artemisinin resistance as well [160].

## Current situation of malaria infection and ongoing studies on antimalarial drug development

Since ACTs have become the major treatment for malaria and strict preventive measures against parasite-infected mosquitoes have been implemented, the malaria-related mortality rate and case incidence have decreased gradually during the past 10 years [1]. Although artemisinin-resistant *Plasmodium* parasites have emerged and show a significant delay in clearance rate, the response of dihydroartemisinin against either wild-type parasites or mutants exhibits similar *K*_m_ values suggesting that dihydroartemisinin does not lose its activity against the mutants [161]. Extending the treatment courses could be an effective strategy to clear resistant parasite infection. However, the parasites can still develop complete resistance against artemisinin-based treatment at any point in the future. In addition, the proportion of malaria-infected patients is concentrated in countries with low national income levels. Among these, more than 68 million infected children do not receive any ACTs [1]. Therefore, large amounts of low-cost artemisinin for ACTs, by either increasing the cultivation of high-artemisinin-producing *A. annua* plants or developing cheaper synthetic biological processes in the long term, are required to prevent any further development of parasites and meet the demand of ACTs worldwide. Moreover, novel effective antimalarial treatments must be developed continually. Recently, low-cost plant-based artemisinin combination therapy (pACT) has driven attention on the production of no semi-synthetic artemisinin *in planta* as this treatment showed higher antimalarial activity, and the synergistic effect of artemisinin and the plant matrix overcame resistance to artemisinin [162–169]. Several scientists have also focused on the investigation of novel potential drug targets [170–180] and on the synthesis of novel antimalarial compounds including artemisinin hybrids [181–186]. Still, further studies on these avenues are required.

## Conclusion

Several approaches to enhance the production of artemisinin have been investigated for over a decade. As a result, the availability of artemisinin for ACTs is increasing, and the number of malaria-related deaths is decreasing gradually. Although artemisinin is still effective against malaria-causing parasites, the emergence of artemisinin-resistant strains has posed a new challenge to scientists worldwide. Therefore, elucidating the mode of action of artemisinin and the mechanism of resistance against this compound in *Plasmodium* parasites is important for further development of antimalarial drugs. We hope that the current understanding of artemisinin as summarized in this review will provide clues for further investigation and development of antimalarial treatments to overcome artemisinin resistance in *Plasmodium* parasites in the future.

## References

[1] World Health Organization (2015). World malaria report 2015.

[2] Tu Y (2011). The discovery of artemisinin (qinghaosu) and gifts from Chinese medicine. Nat Med.

[3] de Ridder S, van der Kooy F, Verpoorte R (2008). *Artemisia annua* as a self-reliant treatment for malaria in developing countries. J Ethnopharmacol.

[4] van Agtmael MA, Eggelte TA, van Boxtel CJ (1999). Artemisinin drugs in the treatment of malaria: from medicinal herb to registered medication. Trends Pharmacol Sci.

[5] Wang J, Zhang CJ, Chia WN, Loh CCY, Li Z, Lee YM, He Y, Yuan LX, Lim TK, Liu M, Liew CX, Lee YQ, Zhang J, Lu N, Lim CT, Hua ZC, Liu B, Shen HM, Tan KSW, Lin Q (2015). Haem-activated promiscuous targeting of artemisinin in *Plasmodium falciparum*. Nat Commun.

[6] Willcox M (2009). *Artemisia* species: from traditional medicines to modern antimalarials—and back again. J Altern Complement Med.

[7] Klonis N, Creek DJ, Tilley L (2013). Iron and heme metabolism in *Plasmodium falciparum* and the mechanism of action of artemisinins. Curr Opin Microbiol.

[8] World Health Organization (2006). Guidelines for the treatment of malaria.

[9] Ferreira JFS, Laughlin JC, Delabays N, de Magalhães PM (2005). Cultivation and genetics of *Artemisia annua* L. for increased production of the antimalarial artemisinin. Plant Genet Resour.

[10] Chang Z (2015). The discovery of qinghaosu (artemisinin) as an effective anti-malaria drug: a unique China story. Sci China Life Sci.

[11] Miller LH, Su X (2011). Artemisinin: discovery from the Chinese herbal garden. Cell.

[12] Su XZ, Miller LH (2015). The discovery of artemisinin and the Nobel Prize in Physiology or Medicine. Sci China Life Sci.

[13] Liao F (2009). Discovery of artemisinin (qinghaosu). Molecules.

[14] World Health Organization (2015). Guidelines for the treatment of malaria.

[15] Li G, Arnold K, Guo X, Jian H, Fu L (1984). Randomised comparative study of mefloquine, qinghaosu, and pyrimethamine–sulfadoxine in patients with falciparum malaria. Lancet.

[16] Brown GD (2010). The biosynthesis of artemisinin (qinghaosu) and the phytochemistry of *Artemisia annua* L. (qinghao). Molecules.

[17] Towler MJ, Weathers PJ (2007). Evidence of artemisinin production from IPP stemming from both the mevalonate and the nonmevalonate pathways. Plant Cell Rep.

[18] Schramek N, Wang H, Römisch-Margl W, Keil B, Radykewicz T, Winzenhörlein B, Beerhues L, Bacher A, Rohdich F, Gershenzon J, Liu B, Eisenreich W (2010). Artemisinin biosynthesis in growing plants of *Artemisia annua*. A ^13^CO_2_ study. Phytochemistry.

[19] Bouwmeester HJ, Wallaart TE, Janssen MHA, van Loo B, Jansen BJM, Posthumus MA, Schmidt CO, De Kraker JW, König WA, Franssen MCR (1999). Amorpha-4,11-diene synthase catalyses the first probable step in artemisinin biosynthesis. Phytochemistry.

[20] Chang YJ, Song SH, Park SH, Kim SU (2000). Amorpha-4,11-diene synthase of *Artemisia annua*: cDNA isolation and bacterial expression of a terpene synthase involved in artemisinin biosynthesis. Arch Biochem Biophys.

[21] Mercke P, Bengtsson M, Bouwmeester HJ, Posthumus MA, Brodelius PE (2000). Molecular cloning, expression and characterization of amorpha-4,11-diene synthase, a key enzyme of artemisinin biosynthesis in *Artemisia annua* L. Arch Biochem Biophys.

[22] Kim SH, Heo K, Chang YJ, Park SH, Rhee SK, Kim SU (2006). Cyclization mechanism of amorpha-4,11-diene synthase, a key enzyme in artemisinin biosynthesis. J Nat Prod.

[23] Picaud S, Mercke P, He X, Sterner O, Brodelius M, Cane DE, Brodelius PE (2006). Amorpha-4,11-diene synthase: mechanism and stereochemistry of the enzymatic cyclization of farnesyl diphosphate. Arch Biochem Biophys.

[24] Teoh KH, Polichuk DR, Reed DW, Nowak G, Covello PS (2006). *Artemisia annua* L. (Asteraceae) trichome-specific cDNAs reveal CYP71AV1, a cytochrome P450 with a key role in the biosynthesis of the antimalarial sesquiterpene lactone artemisinin. FEBS Lett.

[25] Polichuk D, Teoh KH, Zhang Y, Ellens KW, Reed DW, Covello PS (2010) Nucleotide sequence encoding an alcohol dehydrogenase from *Artemisia annua* and uses thereof. Patent No. WO2010/012074

[26] Teoh KH, Polichuk DR, Reed DW, Covello PS (2009). Molecular cloning of an aldehyde dehydrogenase implicated in artemisinin biosynthesis in *Artemisia annua*. Botany.

[27] Brown GD, Sy LK (2007). In vivo transformations of artemisinic acid in *Artemisia annua* plants. Tetrahedron.

[28] Zhang Y, Teoh KH, Reed DW, Maes L, Goossens A, Olson DJH, Ross ARS, Covello PS (2008). The molecular cloning of artemisinic aldehyde Δ11(13) reductase and its role in glandular trichome-dependent biosynthesis of artemisinin in *Artemisia annua*. J Biol Chem.

[29] Brown GD, Sy LK (2004). In vivo transformations of dihydroartemisinic acid in *Artemisia annua* plants. Tetrahedron.

[30] Rydén AM, Ruyter-Spira C, Quax WJ, Osada H, Muranaka T, Kayser O, Bouwmeester H (2010). The molecular cloning of dihydroartemisinic aldehyde reductase and its implication in artemisinin biosynthesis in *Artemisia annua*. Planta Med.

[31] Olofsson L, Engström A, Lundgren A, Brodelius PE (2011). Relative expression of genes of terpene metabolism in different tissues of *Artemisia annua* L. BMC Plant Biol.

[32] Arsenault PR, Vail D, Wobbe KK, Erickson K, Weathers PJ (2010). Reproductive development modulates gene expression and metabolite levels with possible feedback inhibition of artemisinin in *Artemisia annua*. Plant Physiol.

[33] Ma D, Pu G, Lei C, Ma L, Wang H, Guo Y, Chen J, Du Z, Wang H, Li G, Ye H, Liu B (2009). Isolation and characterization of AaWRKY1, an *Artemisia annua* transcription factor that regulates the amorpha-4,11-diene synthase gene, a key gene of artemisinin biosynthesis. Plant Cell Physiol.

[34] Li ZQ, Liu Y, Liu BY, Wang H, Ye HC, Li GF (2006). Cloning, *E. coli* expression and molecular analysis of amorpha-4,11-diene synthase from a high-yield strain of *Artemisia annua* L. J Integr Plant Biol.

[35] Pu GB, Ma DM, Wang H, Ye HC, Liu BY (2013). Expression and localization of amorpha-4,11-diene synthase in *Artemisia annua* L. Plant Mol Biol Rep.

[36] Kim SH, Chang YJ, Kim SU (2008). Tissue specificity and development pattern of amorpha-4,11-diene synthase (ADS) proved by *ADS* promoter-driven GUS expression in the heterologous plant, *Arabidopsis thaliana*. Planta Med.

[37] Wang H, Olofsson L, Lundgren A, Brodelius PE (2011). Trichome-specific expression of amorpha-4,11-diene synthase, a key enzyme of artemisinin biosynthesis in *Artemisia annua* L., as reported by a promoter-GUS fusion. Am J Plant Sci.

[38] Wang Y, Yang K, Jing F, Li M, Deng T, Huang R, Wang B, Wang G, Sun X, Tang KX (2011). Cloning and characterization of trichome-specific promoter of *cpr71av1* gene involved in artemisinin biosynthesis in *Artemisia annua* L. Mol Biol.

[39] Wang H, Han J, Kanagarajan S, Lundgren A, Brodelius PE (2013). Trichome-specific expression of the amorpha-4,11-diene 12-hydroxylase (*cyp71av1*) gene, encoding a key enzyme of artemisinin biosynthesis in *Artemisia annua*, as reported by a promoter-GUS fusion. Plant Mol Biol.

[40] Jiang W, Lu X, Qiu B, Zhang F, Shen Q, Lv Z, Fu X, Yan T, Gao E, Zhu M, Chen L, Zhang L, Wang G, Sun X, Tang K (2014). Molecular cloning and characterization of a trichome-specific promoter of artemisinic aldehyde Δ11(13) reductase (DBR2) in *Artemisia annua*. Plant Mol Biol Rep.

[41] Ting HM, Wang B, Rydén AM, Woittiez L, van Herpen T, Verstappen FWA, Ruyter-Spira C, Beekwilder J, Bouwmeester HJ, van der Krol A (2013). The metabolite chemotype of *Nicotiana benthamiana* transiently expressing artemisinin biosynthetic pathway genes is a function of *CYP71AV1* type and relative gene dosage. New Phytol.

[42] Yang K, Monafared RS, Wang H, Lundgren A, Brodelius PE (2015). The activity of the artemisinic aldehyde Δ11(13) reductase promoter is important for artemisinin yield in different chemotypes of *Artemisia annua* L. Plant Mol Biol.

[43] Sun C, Li J, CaoY Long G, Zhou B (2015). Two distinct and competitive pathways confer the cellcidal actions of artemisinins. Microb Cell.

[44] Wang J, Huang L, Li J, Fan Q, Long Y, Li Y, Zhou B (2010). Artemisinin directly targets malarial mitochondria through its specific mitochondrial activation. PLoS One.

[45] Mercer AE, Copple IM, Maggs JL, O’Neill PM, Park BK (2011). The role of heme and the mitochondrion in the chemical and molecular mechanisms of mammalian cell death induced by the artemisinin antimalarials. J Biol Chem.

[46] Hartwig CL, Rosenthal AS, Angelo JD, Griffin CE, Posner GH, Cooper RA (2009). Accumulation of artemisinin trioxane derivatives within neutral lipids of *Plasmodium falciparum* malaria parasites is endoperoxide-dependent. Biochem Pharmacol.

[47] Antoine T, Fisher N, Amewu R, O’Neill PM, Ward SA, Biagini GA (2014). Rapid kill of malaria parasites by artemisinin and semi-synthetic endoperoxides involves ROS-dependent depolarization of the membrane potential. J Antimicrob Chemother.

[48] O’Neill PM, Barton VE, Ward SA (2010). The molecular mechanism of action of artemisinin—the debate continues. Molecules.

[49] Klonis N, Crespo-Ortiz MP, Bottova I, Abu-Bakar N, Kenny S, Rosenthal PJ, Tilley L (2011). Artemisinin activity against *Plasmodium falciparum* required hemoglobin uptake and digestion. Proc Natl Acad Sci USA.

[50] Xie SC, Dogovski C, Hanssen E, Chiu F, Yang T, Crespo MP, Stafford C, Batinovic S, Teguh S, Charman S, Klonis N, Tilley L (2016). Haemoglobin degradation underpins the sensitivity of early ring stage *Plasmodium falciparum* to artemisinins. J Cell Sci.

[51] Chugh M, Sundararaman V, Kumar S, Reddy VS, Siddiqui WA, Stuart KD, Malhotra P (2013). Protein complex directs hemoglobin-to-hemozoin formation in *Plasmodium falciparum*. Proc Natl Acad Sci USA.

[52] Bhisutthibhan J, Pan XQ, Hossler PA, Walker DJ, Yowell CA, Carlton J, Dame JB, Meshnick SR (1998). The *Plasmodium falciparum* translationally controlled tumor protein homolog and its reaction with the antimalarial drug artemisinin. J Biol Chem.

[53] Eichhorn T, Winter D, Büchele B, Dirdjaja N, Frank M, Lehmann WD, Mertens R, Krauth-Siegel RL, Simmet T, Granzin J, Efferth T (2013). Molecular interaction of artemisinin with translationally controlled tumor protein (TCTP) of *Plasmodium falciparum*. Biochem Pharmacol.

[54] Eckstein-Ludwig U, Webb RJ, van Goethem IDA, East JM, Lee AG, Kimura M, O’Neill PM, Bray PG, Ward SA, Krishna S (2003). Artemisinin target the SERCA of *Plasmodium falciparum*. Nature.

[55] Covello PS (2008). Making artemisinin. Phytochemistry.

[56] Wang Z, Yang L, Yang X, Zhang X (2014). Advances in the chemical synthesis of artemisinin. Synth Commun.

[57] Jain DC, Mathur AK, Gupta MM, Singh AK, Verma RK, Gupta AP, Kumar S (1996). Isolation of high artemisinin-yielding clones of *Artemisia annua*. Phytochemistry.

[58] Delabays N, Simonnet X, Gaudin M (2001). The genetics of artemisinin content in *Artemisia annua* L. and the breeding of high yielding cultivars. Curr Med Chem.

[59] Cockram J, Hill C, Burns C, Arro RRJ, Woolley JG, Flockart I, Robinson T, Atkinson CJ, Davies MJ, Dungey N, Greenland AJ, Smith LLMJ, Bentley S (2012). Screening a diverse collection of *Artemisia annua* germplasm accessions for the antimalarial compound, artemisinin. Plant Gen Res.

[60] Larson TR, Branigan C, Harvey D, Penfield T, Bowles D, Graham IA (2013). A survey of artemisinic and dihydroartemisinic acid contents in glasshouse and global field-grown populations of the artemisinin-producing plant *Artemisia annua* L. Ind Crops Prod.

[61] Graham IA, Besser K, Blumer S, Branigan CA, Czechowski T, Elias L, Guterman I, Harvey D, Isaac PG, Khan AM, Larson TR, Li Y, Pawson T, Penfield T, Rae AM, Rathbone DA, Reid S, Ross J, Smallwood MF, Segura V, Townsend T, Vyas D, Winzer T, Bowles D (2010). The genetic map of *Artemisia annua* L. identifies loci affecting yield of the antimalarial drug artemisinin. Science.

[62] Townsend T, Segura V, Chigeza G, Penfield T, Rae A, Harvey D, Bowles D, Graham IA (2013). The use of combining ability analysis to identify elite parents for *Artemisia annua* F1 hybrid production. PLoS One.

[63] Muranaka T, Saito K, Mander L, Lui HW (2010). Production of pharmaceuticals by plant tissue cultures. Comprehensive natural products II: chemistry and biology. Development and modification of bioactivity.

[64] Weathers PJ, Elkholy S, Wobbe KK (2006). Artemisinin: the biosynthetic pathway and its regulation in *Artemisia annua*, a terpenoid-rich species. In Vitro Cell Dev Biol Plant.

[65] Liu C, Zhao Y, Wang Y (2006). Artemisinin: current state and perspectives for biotechnological production of an antimalarial drug. Appl Microbiol Biotechnol.

[66] Vergauwe A, Cammaert R, Vandenberghe D, Genetelo C, Inze D, Van Montagu M, Van den Eeckhout E (1996). *Agrobacterium tumefaciens*-mediated transformation of *Artemisia annua* L. and regeneration of transgenic plants. Plant Cell Rep.

[67] Vergauwe A, Van Geldre E, Inzé D, Van Montagu M, Van den Eeckhout E (1998). Factors influencing *Agrobacterium tumefaciens*-mediated transformation of *Artemisia annua* L. Plant Cell Rep.

[68] Han JL, Wang H, Ye HC, Liu Y, Li ZQ, Zhang Y, Zhang YS, Yan F, Li GF (2005). High efficiency of genetic transformation and regeneration of *Artemisia annua* L. via *Agrobacterium tumefaciens*-mediated procedure. Plant Sci.

[69] Lualon W, De-Eknamkul W, Tanaka H, Shoyama Y, Putalun W (2008). Artemisinin production by shoot regeneration of *Artemisia annua* L. using thidiazuron. Z Naturforsch.

[70] Tian N, Liu S, Ting H, Huang J, van der Krol S, Bouwmeester H, Liu Z (2013). An improved *Agrobacterium tumefaciens* mediated transformation of *Artemisia annua* L. by using stem internodes as explants. Czech J Genet Plant Breed.

[71] Wang J, Nie J, Pattanaik S, Yuan L (2016). Efficient *Agrobacterium*-mediated transformation of *Artemisia annua* L. using young inflorescence. In Vitro Cell Dev Biol.

[72] Kiani BH, Suberu J, Barker GC, Mirza B (2014). Development of efficient miniprep transformation methods for *Artemisia annua* using *Agrobacterium tumefaciens* and *Agrobacterium rhizogenes*. In Vitro Cell Dev Biol.

[73] Nguyen KT, Towler MJ, Weathers PJ (2013). The effect of roots and media constituents on trichomes and artemisinin production in *Artemisia annua* L. Plant Cell Rep.

[74] Chen DH, Ye HC, Li GF (2000). Expression of a chimeric farnesyl diphosphate synthase gene in *Artemisia annua* L. transgenic plants via *Agrobacterium tumefaciens*-mediated transformation. Plant Sci.

[75] Banyai W, Kirdmanee C, Mii M, Supaibulwatana K (2010). Overexpression of farnesyl pyrophosphate synthase (*FPS*) gene affected artemisinin content and growth of *Artemisia annua* L. Plant Cell Tissue Organ Cult.

[76] Shen Q, Chen YF, Wang T, Wu SY, Lu X, Zhang L, Zhang FY, Jiang WM, Wang GF, Tang KX (2012). Overexpression of the cytochrome P450 monooxygenase (*cyp71av1*) and cytochrome P450 reductase (*cpr*) genes increased artemisinin content in *Artemisia annua* (Asteraceae). Gen Mol Res.

[77] Xiang L, Zeng L, Yuan Y, Chen M, Wang F, Liu X, Zeng L, Lan X, Liao Z (2012). Enhancement of artemisinin biosynthesis by overexpressing *dxr*, *cyp71av1* and *cpr* in the plants of *Artemisia annua* L. Plant Omics J.

[78] Yuan Y, Liu W, Zhang Q, Xiang L, Liu X, Chen M, Lin Z, Wang Q, Liao Z (2015). Overexpression of artemisinic aldehyde Δ11(13) reductase gene-enhanced artemisinin and its relative metabolite biosynthesis in transgenic *Artemisia annua* L. Biotechnol Appl Biochem.

[79] Chen Y, Shen Q, Wang Y, Wang T, Wu S, Zhang L, Lu X, Zhang F, Jiang W, Qiu B, Gao E, Sun X, Tang K (2013). The stacked over-expression of *FPS*, *CYP71AV1* and *CPR* genes leads to the increase of artemisinin level in *Artemisia annua* L. Plant Biotechnol Rep.

[80] Wang Y, Jing F, Yu S, Chen Y, Wang T, Liu P, Wang G, Sun X, Tang K (2011). Co-overexpression of the *HMGR* and *FPS* genes enhances artemisinin content in *Artemisia annua* L. J Med Plant Res.

[81] Alam P, Abdin MZ (2011). Over-expression of HMG-CoA reductase and amorpha-4,11-diene synthase genes in *Artemisia annua* L. and its influence on artemisinin content. Plant Cell Rep.

[82] Alam P, Kamaludding Sharaf-Eldin MA, Elkholy SF, Abdin MZ (2015). The effect of over-expression of rate limiting enzymes on the yield of artemisinin in *Artemisia annua*. Rend Fis Acc Lincei.

[83] Zhang L, Jin F, Li F, Li M, Wang Y, Wang G, Sun X, Tang K (2009). Development of transgenic *Artemisia annua* (Chinese wormwood) plants with an enhanced content of artemisinin, an effective anti-malarial drug, by hairpin-RNA-mediated gene silencing. Biotechnol Appl Biochem.

[84] Ferreira JFS (2007). Nutrient deficiency in the production of artemisinin, dihydroartemisinic acid, and artemisinic acid in *Artemisia annua* L. J Agric Food Chem.

[85] Wang ML, Jiang YS, Wei JQ, Wei X, Qi XX, Jiang SY, Wang ZM (2007). Effects of irradiance on growth, photosynthetic characteristics, and artemisinin content of *Artemisia annua* L. Photosynthetica.

[86] Pan WS, Zheng LP, Tian H, Li WY, Wan JW (2014). Transcriptome responses involved in artemisinin production in *Artemisia annua* L. under UV-B radiation. J Photochem Photobiol B.

[87] Marchese JA, Ferreira JFS, Rehder VLG, Rodrigues O (2010). Water deficit effect on the accumulation of biomass and artemisinin in annual wormwood (*Artemisia annua* L., Asteraceae). Braz J Plant Physiol.

[88] Nguyen KT, Arsenault PR, Weathers PJ (2011). Trichomes + roots + ROS = artemisinin: regulating artemisinin biosynthesis in *Artemisia annua* L. In Vitro Cell Dev Biol Plant.

[89] Pandey N, Pandey-Rai S (2016). Updates on artemisinin: an insight to mode of actions and strategies for enhanced global production. Protoplasma.

[90] Han J, Wang H, Lundgren A, Brodelius PE (2014). Effects of overexpression of *AaWRKY1* on artemisinin biosynthesis in transgenic *Artemisia annua* plants. Phytochemistry.

[91] Lu X, Zhang L, Zhang F, Jiang W, Shen Q, Zhang L, Lv Z, Wang G, Tang K (2013). *AaORA*, a trichome-specific AP2/ERF transcription factor of *Artemisia annua*, is a positive regulator in the artemisinin biosynthetic pathway and in disease resistance to *Botrytis cinerea*. New Phytol.

[92] Yu ZX, Li JX, Yang CQ, Hu WL, Wang LJ, Chen XY (2012). The jasmonate-responsive AP2/ERF transcription factors AaERF1 and AaERF2 positively regulate artemisinin biosynthesis in *Artemisia annua* L. Mol Plant.

[93] Ji Y, Xiao J, Shen Y, Ma D, Li Z, Pu G, Li X, Huang L, Liu B, Ye H, Wang H (2014). Cloning and characterization of AabHLH1, a bHLH transcription factor that positively regulates artemisinin biosynthesis in *Artemisia annua*. Plant Cell Physiol.

[94] Zhang F, Fu X, Lv Z, Lu X, Shen Q, Zhang L, Zhu M, Wang G, Sun X, Liao Z, Tang K (2015). A basic leucine zipper transcription factor, AabZIP1, connects abscisic acid signaling with artemisinin biosynthesis in *Artemisia annua*. Mol Plant.

[95] Pu GB, Ma DM, Chen JL, Ma LQ, Wang H, Li GF, Ye HC, Liu BY (2009). Salicylic acid activates artemisinin biosynthesis in *Artemisia annua* L. Plant Cell Rep.

[96] Maes L, Van Nieuwerburgh FCW, Zhang Y, Reed DW, Pollier J, Vande Casteele SRF, Inzé D, Covello PS, Deforce DLD, Goossens A (2011). Dissection of the phytohormonal regulation of trichome formation and biosynthesis of the antimalarial compound artemisinin in *Artemisia annua* plants. New Phytol.

[97] Caretto S, Quarta A, Durante M, Nisi R, De Paolis A, Blando F, Mita G (2011). Methyl jasmonate and miconazole differently affect artemisinin production and gene expression in *Artemisia annua* suspension cultures. Plant Biol.

[98] Xiang L, Zhu S, Zhao T, Zhang M, Liu W, Chen M, Lan X, Liao Z (2015). Enhancement of artemisinin content and relative expression of genes of artemisinin biosynthesis in *Artemisia annua* by exogenous MeJA treatment. Plant Growth Regul.

[99] Zhang L, Lu X, Shen Q, Chen Y, Wang T, Zhang F, Wu S, Jiang W, Liu P, Zhang L, Wang Y, Tang K (2012). Identification of putative *Artemisia annua* ABCG transporter unigenes related to artemisinin yield following expression analysis in different plant tissues and in response to methyl jasmonate and abscisic acid treatments. Plant Mol Biol Rep.

[100] Lu X, Lin X, Shen Q, Zhang F, Wang Y, ChenY Wang T, Wu S, Tang K (2011). Characterization of the jasmonate biosynthetic gene allene oxide cyclase in *Artemisia annua* L., source of the antimalarial drug artemisinin. Plant Mol Biol Rep.

[101] Zhang F, Lu X, Lv Z, Zhang L, Zhu M, Jiang W, Wang G, Sun X, Tang K (2013). Overexpression of the *Artemisia* orthologue of ABA receptor, AaPYL9, enhances ABA sensitivity and improves artemisinin content in *Artemisia annua* L. PLoS One.

[102] Singh ND, Kumar S, Daniell H (2016). Expression of β-glucosidase increases trichome density and artemisinin content in transgenic *Artemisia annua* plants. Plant Biotechnol J.

[103] Dilshad E, Cusido RM, Palazon J, Estrada KR, Bonfill M, Mirza B (2015). Enhanced artemisinin yield by expression of *rol* genes in *Artemisia annua*. Malar J.

[104] Arora M, Saxena P, Choudhary DK, Abdin MZ, Varma A (2016). Dual symbiosis between *Piriformospora indica* and *Azotobacter chroococcum* enhances the artemisinin content in *Artemisia annua* L. World J Microbiol Biotechnol.

[105] Awasthi A, Bharti N, Nair P, Singh R, Shukla AK, Gupta MM, Darokar MP, Kalra A (2011). Synergistic effect of *Glomus mosseae* and nitrogen fixing *Bacillus subtilis* strain Daz26 on artemisinin content in *Artemisia annua* L. Appl Soil Ecol.

[106] van Herpen TWJM, Cankar K, Nogueira M, Bosch D, Bouwmeester HJ, Beekwilder J (2010). *Nicotiana benthamiana* as a production platform for artemisinin precursors. PLoS One.

[107] Saxena B, Subramaniyan M, Malhotra K, Bhavesh NS, Potlakayala SD, Kumar S (2014). Metabolic engineering of chloroplasts for artemisinic acid biosynthesis and impact on plant growth. J Biosci.

[108] Marienhagen J, Bott M (2013). Metabolic engineering of microorganisms for the synthesis of plant natural products. J Biotechnol.

[109] Martin VJJ, Pitera DJ, Withers ST, Newman JD, Keasling JD (2003). Engineering a mevalonate pathway in *Escherichia coli* for production of terpenoids. Nat Biotechnol.

[110] Newman JD, Marshall J, Chang M, Nowroozi F, Paradise E, Pitera D, Newman KL, Keasling JD (2006). High-level production of amorpha-4,11-diene in a two-phase partitioning bioreactor of metabolically engineered *Escherichia coli*. Biotechnol Bioeng.

[111] Pitera DJ, Paddon CJ, Newman JD, Keasling JD (2007). Balancing a heterologous mevalonate pathway for improved isoprenoid production in *Escherichia coli*. Metab Eng.

[112] Anthony JR, Anthony LC, Nowroozi F, Kwon G, Newman JD, Keasling JD (2009). Optimization of the mevalonate-based isoprenoid biosynthetic pathway in *Escherichia coli* for production of the anti-malarial drug precursor amorpha-4,11-diene. Metab Eng.

[113] Tsuruta H, Paddon CJ, Eng D, Lenihan JR, Horning T, Anthony LC, Regentin R, Keasling JD, Renninger NS, Newman JD (2009). High-level production of amorpha-4,11-diene, a precursor of the antimalarial agent artemisinin, in *Escherichia coli*. PLoS One.

[114] Zhang C, Chen X, Zou R, Zhou K, Stephanopoulos G, Too HP (2013). Combining genotype improvement and statistical media optimization for isoprenoid production in *E. coli*. PLoS One.

[115] Zhang C, Zou R, Chen X, Stephanopoulos G, Too HP (2015). Experimental design-aided systematic pathway optimization of glucose uptake and deoxyxylulose phosphate pathway for improved amorphadiene production. Appl Microbiol Biotechnol.

[116] Wang JF, Xiong ZQ, Li SY, Wang Y (2013). Enhancing isoprenoid production through systematically assembling and modulating efflux pumps in *Escherichia coli*. Appl Microbiol Biotechnol.

[117] Zhang C, Chen X, Stephanopoulos G, Too HP (2016). Efflux transporter engineering markedly improves amorphadiene production in *Escherichia coli*. Biotechnol Bioeng.

[118] Chang MCY, Eachus RA, Trieu W, Ro DK, Keasling JD (2007). Engineering *Escherichia coli* for production of functionalized terpenoids using plant P450s. Nat Chem Biol.

[119] Dietrich JA, Yoshikuni Y, Fisher KJ, Woolard FX, Ockey D, McPhee DJ, Renninger NS, Chang MCY, Baker D, Keasling JD (2009). A novel semi-biosynthetic route for artemisinin production using engineered substrate-promiscuous P450_BM3_. ACS Chem Biol.

[120] Lindahl AL, Olsson ME, Mercke P, Tollbom O, Schelin J, Brodelius M, Brodelius PE (2006). Production of the artemisinin precursor amorpha-4,11-diene by engineered *Saccharomyces cerevisiae*. Biotechnol Lett.

[121] Ro DK, Paradise EM, Ouellet M, Fisher KJ, Newman KL, Ndungu JM, Ho KA, Eachus RA, Ham TS, Kirby J, Chang MCY, Withers ST, Shiba Y, Sarpong R, Keasling JD (2006). Production of the antimalarial drug precursor artemisinic acid in engineered yeast. Nature.

[122] Ro DK, Ouellet M, Paradise EM, Burd H, Eng D, Paddon CJ, Newman JD, Keasling JD (2008). Induction of multiple pleiotropic drug resistance genes in yeast engineered to produce an increased level of anti-malarial drug precursor, artemisinic acid. BMC Biotechnol.

[123] Lenihan JR, Tsuruta H, Diola D, Renninger NS, Regentin R (2008). Developing an industrial artemisinic acid fermentation process to support the cost-effective production of antimalarial artemisinin-based combination therapies. Biotechnol Prog.

[124] Westfall PJ, Pitera DJ, Lenihan JR, EngD Woolard FX, Regentin R, Horning T, Tsuruta H, Melis DJ, Owens A, Fickes S, Diola D, Benjamin KR, Keasling JD, Leavell MD, McPhee DJ, Renninger NS, Newman JD, Paddon CJ (2012). Production of amorphadiene in yeast, and its conversion to dihydroartemisinic acid, precursor to the antimalarial agent artemisinin. Proc Natl Acad Sci USA.

[125] Schenkman JB, Jansson I (2003). The many roles of cytochrome *b*_5_. Pharmacol Ther.

[126] Paddon CJ, Westfall PJ, Pitera DJ, Benjamin K, Fisher K, McPhee D, Leavell MD, Tai A, Main A, Eng D, Polichuk DR, Teoh KH, Reed DW, Treynor T, Lenihan J, Fleck M, Bajad S, Dang G, Diola D, Dorin G, Ellens KW, Fickes S, Galazzo J, Gaucher SP, Geistlinger T, Henry R, Hepp M, Horning T, Iqbal T, Jiang H, Kizer L, Lieu B, Melis D, Moss N, Regentin R, Secrest S, Tsuruta H, Vazquez R, Westblade LF, Xu L, Yu M, Zhang Y, Zhao L, Lievense J, Covello PS, Keasling JD, Reiling KK, Renninger NS, Newman JD (2013). High-level semi-synthetic production of the potent antimalarial artemisinin. Nature.

[127] Paddon CJ, Keasling JD (2014). Semi-synthetic artemisinin: a model for the use of synthetic biology in pharmaceutical development. Nat Rev.

[128] Horváth Z, Horosanskaia E, Lee JW, Lorenz H, Gilmore K, Seeberger PH, Seidel-Morgenstern A (2015). Recovery of artemisinin from a complex reaction mixture using continuous chromatography and crystallization. Org Process Res Dev.

[129] Mannan A, Shaheen N, Arshad W, Qureshi RA, Zia M, Mirza B (2008). Hairy roots induction and artemisinin analysis in *Artemisia dubia* and *Artemisia indica*. Afr J Biotechnol.

[130] Mannan A, Ahmed I, Arshad W, Asim MF, Qureshi RA, Hussain I, Mirza B (2010). Survey of artemisinin production by diverse *Artemisia* species in northern Pakistan. Malar J.

[131] Mannan A, Ahmed I, Arshad W, Hussain I, Mirza B (2011). Effects of vegetative and flowering stages on the biosynthesis of artemisinin in *Artemisia* species. Arch Pharm Res.

[132] Arab HA, Rahbari S, Rassouli A, Moslemi MH, Khosravirad F (2006). Determination of artemisinin in *Artemisia sieberi* and anticoccidial effects of the plant extract in broiler chickens. Trop Anim Health Prod.

[133] Zia M, Mannan A, Chaudhary MF (2007). Effect of growth regulators and amino acids on artemisinin production in the callus of *Artemisia absinthium*. Pak J Bot.

[134] Dilshad E, Cusido RM, Estrada KR, Bonfill M, Mirza B (2015). Genetic transformation of *Artemisia carvifolia* Buch with *rol* genes enhances artemisinin accumulation. PLoS One.

[135] Nibret E, Wink M (2009). Volatile components of four Ethiopian *Artemisia* species extracts and their in vitro antitrypanosomal and cytotoxic activities. Phytomedicine.

[136] Kraft C, Jenett-Siems K, Siems K, Jakupovic J, Mavi S, Bienzle U, Eich E (2003). In vitro antiplasmodial evaluation of medicinal plants from Zimbabwe. Phytother Res.

[137] Gathirwa JW, Rukunga GM, Njagi ENM, Omar SA, Guantai AN, Muthaura CN, Mwitari PG, Kimani CW, Kirira PG, Tolo FM, Ndunda TN, Ndiege IO (2007). In vitro anti-plasmodial and in vivo anti-malarial activity of some plants traditionally used for the treatment of malaria by the Meru community in Kenya. J Nat Med.

[138] Ramazani A, Sardari S, Zakeri S, Vaziri B (2010). In vitro antiplasmodial and phytochemical study of five *Artemisia* species from Iran and in vivo activity of two species. Parasitol Res.

[139] Komori A, Suzuki M, Seki H, Nishizawa T, Meyer JJM, Shimizu H, Yokoyama S, Muranaka T (2013). Comparative functional analysis of CYP71AV1 natural variants reveals an important residue for the successive oxidation of amorpha-4,11-diene. FEBS Lett.

[140] Muangphrom P, Suzuki M, Seki H, Fukushima EO, Muranaka T (2014). Functional analysis of orthologous artemisinic aldehyde Δ11(13)-reductase reveals potential artemisinin-producing activity in non-artemisinin-producing *Artemisia absinthium*. Plant Biotechnol.

[141] Dondorp AM, Nosten F, Yi P, Das D, Phyo AP, Tarning J, Lwing KM, Ariey F, Hanpithakpong W, Lee SJ, Ringwald P, Silamut K, Imwong M, Chotivanich K, Lim P, Herdman T, An SS, Yeung S, Singhasivanon P, Day NPJ, Lindegardh N, Socheat D, White NJ (2009). Artemisinin resistance in *Plasmodium falciparum* malaria. N Engl J Med.

[142] Witkowski B, Khim N, Chim P, Kim S, Ke S, Kloeung N, Chy S, Duong S, Leang R, Ringwald P, Dondorp AM, Tripura R, Benoit-Vical F, Berry A, Gorgette O, Ariey F, Barale JC, Mercereau-Puijalon O, Menard D (2013). Reduced artemisinin susceptibility of *Plasmodium falciparum* ring stages in western Cambodia. Antimicrob Agents Chemother.

[143] Ashley EA, Dhorda M, Fairhurst RM, Amaratunga C, Lim P, Suon S, Sreng S, Anderson JM, Mao S, Sam B, Sopha C, Chuor CM, Nguon C, Sovannaroth S, Pukrittayakamee S, Jittamala P, Chotivanich K, Chutasmit K, Suchatsoonthorn C, Runcharoen R, Hien TT, Thuy-Nhien NT, Thanh NV, Phu NH, Htut Y, Han KT, Aye KH, Mokuolu OA, Olaosebikan RR, Folaranmi OO, Mayxay M, Khanthavong M, Hongvanthong B, Newton PN, Onyamboko MA, Fanello CI, Tshefu AK, Mishra N, Valecha N, Phyo AP, Nosten F, Yi P, Tripura R, Borrmann S, Bashraheil M, Peshu J, Faiz MA, Ghose A, Hossain MA, Samad R, Rahman MR, Hasan MM, Islam A, Miotto O, Amato R, MacInnis B, Stalker J, Kwiatkowski DP, Bozdech Z, Jeeyapant A, Cheah PY, Sakulthaew T, Chalk J, Intharabut B, Silamut K, Lee SJ, Vihokhern B, Kunasol C, Imwong M, Tarning J, Taylor WJ, Yeung S, Woodrow CJ, Flegg JA, Das D, Smith J, Venkatesan M, Plowe CV, Stepniewska K, Guerin PJ, Dondorp AM, Day NP, White NJ (2014). Spread of artemisinin resistance in *Plasmodium falciparum* malaria. N Engl J Med.

[144] Amaratunga C, Sreng S, Suon S, Phelps ES, Stepniewska K, Lim P, Zhou C, Mao S, Anderson JM, Lindegardh N, Jiang H, Song J, Su XZ, White NJ, Dondorp AM, Anderson TJC, Fay MP, Mu J, Duong S, Fairhurst RM (2012). Artemisinin-resistant *Plasmodium falciparum* in Pursat province, western Cambodia: a parasite clearance rate study. Lancet Infect Dis.

[145] Afonso A, Hunt P, Cheesman S, Alves AC, Cunha CV, do Rosário V, Cravo P (2006). Malaria parasites can develop stable resistance to artemisinin but lack mutations in candidate genes *atp6* (encoding the sarcoplasmic and endoplasmic reticulum Ca^2+^ ATPase), *tctp*, *mdr1*, and *cg10*. Antimicrob Agents Chemother.

[146] Witkowski B, Amaratunga C, Khim N, Sreng S, Chim P, Kim S, Lim P, Mao S, Sopha C, Sam B, Anderson JM, Duong S, Chuor CM, Taylor WRJ, Suon S, Mercereau-Puijalon O, Fairhurst RM, Menard D (2013). Novel phenotypic assays for the detection of artemisinin-resistant *Plasmodium falciparum* malaria in Cambodia: in vitro and ex vivo drug-response studies. Lancet Infect Dis.

[147] Mok S, Ashley EA, Ferreira PE, Zhu L, Lin Z, Yeo T, Chotivanich K, Imwong M, Pukrittayakamee S, Dhorda M, Nguon C, Lim P, Amaratunga C, Suon S, Hien TT, Htut Y, Faiz MA, Onyamboko MA, Mayxay M, Newton PN, Tripura R, Woodrow CJ, Miotto O, Kwiatkowski DP, Nosten F, Day NPJ, Preiser PR, White NJ, Dondorp AM, Fairhurst RM, Bozdech Z (2015). Population transcriptomics of human malaria parasites reveals the mechanism of artemisinin resistance. Science.

[148] Takala-Harrison S, Clark TG, Jacob CG, Cummings MP, Miotto O, Dondorp AM, Fukuda MM, Nosten F, Noedl H, Imwong M, Bethell D, Se Y, Lon C, Tyner SD, Saunders DL, Socheat D, Ariey F, Phyo AP, Starzengruber P, Fuehrer HP, Swoboda P, Stepniewska K, Flegg J, Arze C, Cerqueira GC, Silva JC, Ricklefs SM, Porcella SF, Stephens RM, Adams M, Kenefic LJ, Campino S, Auburn S, MacInnis B, Kwiatkowski DP, Su XZ, White NJ, Ringwald P, Plowe CV (2012). Genetic loci associated with delayed clearance of *Plasmodium falciparum* following artemisinin treatment in Southeast Asia. Proc Natl Acad Sci USA.

[149] Cheeseman IH, Miller BA, Nair S, Nkhoma S, Tan A, Tan JC, Saai SA, Phyo AP, Moo CL, Lwing KM, McGready R, Ashley E, Imwong M, Stepniewska K, Yi P, Dondorp AM, Mayxay M, Newton PN, White NJ, Nosten F, Ferdig MT, Anderson TJC (2012). A major genome region underlying artemisinin resistance in Malaria. Science.

[150] Miotto O, Almagro-Garcia J, Manske M, MacInnis B, Campino S, Rockett KA, Amaratunga C, Lim P, Suon S, Sreng S, Anderson JM, Duong S, Nguon C, Chuor CM, Saunders D, Se Y, Lon C, Fukuda MM, Amenga-Etego L, Hodgson AVO, Asoala V, Imwong M, Takala-Harrison S, Nosten F, Su XZ, Ringwald P, Ariey F, Dolecek C, Hien TT, Boni MF, Thai CQ, Amambua-Ngwa A, Conway DJ, Djimdé AA, Doumbo OK, Zongo I, Ouedraogo JB, Alcock D, Drury E, Auburn S, Koch O, Sanders M, Hubbart C, Maslen G, Ruano-Rubio V, Jyothi D, Miles A, O’Brien J, Gamble C, Oyola SO, Rayner JC, Newbold CI, Berriman M, Spencer CCA, McVean G, Day NP, White NJ, Bethell D, Dondorp AM, Plowe CV, Fairhurst RM, Kwiatkowski DP (2013). Multiple populations of artemisinin-resistant *Plasmodium falciparum* in Cambodia. Nat Genet.

[151] Ariey F, Witkowski B, Amaratunga C, Beghain J, Langlois AC, Khim N, Kim S, Duru V, Bouchier C, Ma L, Lim P, Leang R, Duong S, Sreng S, Suon S, Chuor CM, Bout DM, Ménard S, Rogers WO, Genton B, Fandeur T, Miotto O, Ringwald P, Le Bras J, Berry A, Barale JC, Fairhurst RM, Benoit-Vical F, Mercereau-Puijalon O, Ménard D (2014). A molecular marker of artemisinin-resistant *Plasmodium falciparum* malaria. Nature.

[152] Isozumi R, Uemura H, Kimata I, Ichinose Y, Logedi J, Omar AH, Kaneko A (2015). Novel mutations in K13 propeller gene of artemisinin-resistant *Plasmodium falciparum*. Emerg Infect Dis.

[153] Straimer J, Gnädig NF, Witkowski B, Amaratunga C, Duru V, Ramadani AP, Dacheux M, Khim N, Zhang L, Lam S, Gregory PD, Urnov FD, Mercereau-Puijalon O, Benoit-Vical F, Fairhurst RM, Ménard D, Fidock DA (2015). K13-propeller mutations confer artemisinin resistance in *Plasmodium falciparum* clinical isolates. Science.

[154] Nyunt MH, Hlaing T, Oo HW, Tin-Oo LLK, Phway HP, Wang B, Zaw NN, Han SS, Tun T, San KK, Kyaw MP, Han ET (2015). Molecular assessment of artemisinin resistance markers, polymorphisms in the K13 propeller, and a multidrug-resistance gene in the eastern and western border areas of Myanmar. Clin Infect Dis.

[155] Bayih AG, Getnet G, Alemu A, Getie S, Mohon AN, Pillai DR (2016). A unique *Plasmodium falciparum Kelch 13* gene mutation in northwest Ethiopia. Am J Trop Med Hyg.

[156] Mbengue A, Bhattacharjee S, Pandharkar T, Liu H, Estiu G, Stahelin RV, Rizk SS, Njimoh DL, Ryan Y, Chotivanich K, Nguon C, Ghorbal M, Lopez-Rubio JJ, Pfrender M, Emrich S, Mohandas N, Dondorp AM, Wiest O, Haldar K (2015). A molecular mechanism of artemisinin resistance in *Plasmodium falciparum* malaria. Nature.

[157] Mita T, Tachibana S, Hashimoto M, Hirai M (2016). *Plasmodium falciparum kelch 13*: a potential molecular marker for tackling artemisinin-resistant malaria parasites. Expert Rev Anti-Infect Ther.

[158] Wang Z, Wang Y, Cabrera M, Zhang Y, Gupta B, Wu Y, Kemirembe K, Hu Y, Liang X, Brashear A, Shrestha S, Li X, Miao J, Sun X, Yang Z, Cui L (2015). Artemisinin resistance at the China–Myanmar border and association with mutations in the K13 propeller gene. Antimicrob Agents Chemother.

[159] Humphreys GS, Merinopoulos I, Ahmed J, Whitty CJM, Mutabingwa TK, Sutherland CJ, Hallett RL (2007). Amodiaquine and artemether–lumefantrine select distinct alleles of the *Plasmodium falciparum mdr1* gene in Tanzanian children treated for uncomplicated malaria. Antimicrob Agents Chemother.

[160] van der Velden M, Rijpma SR, Russel FGM, Sauerwein RW, Koenderink JB (2015). *Pf*MDR2 and *Pf*MDR5 are dispensable for *Plasmodium falciparum* asexual parasite multiplication but change in vitro susceptibility to anti-malarial drugs. Malar J.

[161] Dogovski C, Xie SC, Burgio G, Bridgford J, Mok S, McCaw JM, Chotivanich K, Kenny S, Gnädig N, Straimer J, Bozdech Z, Fidock DA, Simpson JA, Dondorp AM, Foote S, Klonis N, Tilley L (2015). Targeting the cell stress response of *Plasmodium falciparum* to overcome artemisinin resistance. PLoS Biol.

[162] Elfawal MA, Towler MJ, Reich NG, Weathers PJ, Golenbock D, Rich SM (2012). Dried whole plant *Artemisia annua* as an antimalarial therapy. PLoS One.

[163] Onimus M, Carteron S, Lutgen P (2013). The surprising efficiency of *Artemisia annua* powder capsules. Med Aromat Plants.

[164] Weathers PJ, Elfawal MA, Towler MJ, Acquaah-Mensah GK, Rich SM (2014). Pharmacokinetics of artemisinin delivered by oral consumption of *Artemisia annua* dried leaves in healthy vs. *Plasmodium chabaudi*-infected mice. J Ethnopharmacol.

[165] Weathers PJ, Jordan NJ, Lasin P, Towler MJ (2014). Simulated digestion of dried leaves of *Artemisia annua* consumed as a treatment (pACT) for malaria. J Ethnopharmacol.

[166] Weathers PJ, Towler MJ (2014). Changes in key constituents of clonally propagated *Artemisia annua* L. during preparation of compressed leaf tablets for possible therapeutic use. Ind Crops Prod.

[167] Weathers PJ, Towler MJ, Hassanali A, Lutgen P, Engeu PO (2014). Dried-leaf *Artemisia annua*: a practical malaria therapeutic for developing countries?. World J Pharmacol.

[168] Towler MJ, Weathers PJ (2015). Variations in key artemisinic and other metabolites throughout plant development in *Artemisia** annua* L. for potential therapeutic use. Ind Crops Prod.

[169] Elfawal MA, Towler MJ, Reich NG, Weathers PJ, Rich SM (2015). Dried whole-plant *Artemisia annua* slows evolution of malaria drug resistance and overcomes resistance to artemisinin. Proc Natl Acad Sci USA.

[170] Giganti D, Bouillon A, Tawk L, Robert F, Martinez M, Crublet E, Weber P, Girard-Blanc C, Petres S, Haouz A, Hernandez JF, Mercereau-Puijalon O, Alzari PM, Barale JC (2014). A novel *Plasmodium*-specific prodomain fold regulates the malaria drug target SUB1 subtilase. Nat Commun.

[171] Guggisberg AM, Park J, Edwards RL, Kelly ML, Hodge DM, Tolia NH, Odom AR (2014). A sugar phosphatase regulates the methylerythritol phosphate (MEP) pathway in malaria parasites. Nat Commun.

[172] Wright MH, Clough B, Rackham MD, Rangachari K, Brannigan JA, Grainger M, Moss DK, Bottrill AR, Heal WP, Broncel M, Serwa RA, Brady D, Mann DJ, Leatherbarrow RJ, Tewari R, Wilkinson AJ, Holder AA, Tate EW (2013). Validation of *N*-myristoyltransferase as an antimalarial drug target using an integrated chemical biology approach. Nat Chem.

[173] Allen SM, Lim EE, Jortzik E, Preuss J, Chua HH, MacRae JI, Rahlfs S, Haeussler K, Downton MT, McConville MJ, Becker K, Ralph SA (2015). *Plasmodium falciparum* glucose-6-phosphate dehydrogenase 6-phosphogluconolactonase is a potential drug target. FEBS J.

[174] Li H, van der Linden WA, Verdoes M, Florea BI, McAllister FE, Govindaswamy K, Elias JE, Bhanot P, Overkleeft HS, Bogyo M (2014). Assessing subunit dependency of the *Plasmodium* proteasome using small molecule inhibitors and active site probes. ACS Chem Biol.

[175] Yuthavong Y, Tarnchompoo B, Vilaivan T, Chitnumsub P, Kamchonwongpaisan S, Charman SA, McLennan D, White KL, Vivas L, Bongard E, Thongphanchang C, Taweechai S, Vanichtanankul J, Rattanajak R, Arwon U, Fantauzzi P, Yuvaniyama J, Charman WN, Matthews D (2012). Malarial dihydrofolate reductase as a paradigm for drug development against a resistance-compromised target. Proc Natl Acad Sci USA.

[176] Mokmak W, Chunsrivirot S, Hannongbua S, Yuthavong Y, Tongsima S, Kamchonwongpaisan S (2014). Molecular dynamics of interactions between rigid and flexible antifolates and dihydrofolate reductase from pyrimethamine-sensitive and pyrimethamine-resistant *Plasmodium falciparum*. Chem Biol Drug Des.

[177] Chitnumsub P, Jaruwat A, Riangrungroj P, Ittarat W, Noytanom K, Oonanant W, Vanichthanankul J, Chuankhayan P, Maenpuen S, Chen CJ, Chaiyen P, Yuthavong Y, Leartsakulpanich U (2014). Structures of *Plasmodium vivax* serine hydroxymethyltransferase: implications for ligand-binding specificity and functional control. Acta Crystallogr D Biol Crystallogr.

[178] Pinthong C, Maenpuen S, Amornwatcharapong W, Yuthavong Y, Leartsakulpanich U, Chaiyen P (2014). Distinct biochemical properties of human serine hydroxymethyltransferase compared with the *Plasmodium* enzyme: implications for selective inhibition. FEBS J.

[179] Chitnumsub P, Ittarat W, Jaruwat A, Noytanom K, Amornwatcharapong W, Pomthanakasem W, Chaiyen P, Yuthavong Y, Leartsakulpanich U (2014). The structure of *Plasmodium falciparum* serine hydroxymethyltransferase reveals a novel redox switch that regulates its activities. Acta Crystallogr D Biol Crystallogr.

[180] Maenpuen S, Amornwatcharapong W, Krasatong P, Sucharitakul J, Palfey BA, Yuthavong Y, Chitnumsub P, Leartsakulpanich U, Chaiyen P (2015). Kinetic mechanism and the rate-limiting step of *Plasmodium vivax* serine hydroxymethyltransferase. J Biol Chem.

[181] Henrich PP, O’Brien C, Sáenz FE, Cremers S, Kyle DE, Fidock DA (2014). Evidence for pyronaridine as a highly effective partner drug for treatment of artemisinin-resistant malaria in a rodent model. Antimicrob Agents Chemother.

[182] Sun W, Tanaka TQ, Magle CT, Huang W, Southall N, Huang R, Dehdashti SJ, McKew JC, Williamson KC, Zheng W (2014). Chemical signatures and new drug targets for gametocytocidal drug development. Sci Rep.

[183] Singh C, Verma VP, Hassam M, Singh AS, Naikade NK, Puri SK (2014). New orally active amino- and hydroxy-functionalized 11-azaartemisinins and their derivatives with high order of antimalarial activity against multidrug-resistant *Plasmodium yoelii* in Swiss mice. J Med Chem.

[184] Oliveira R, Newton AS, Guedes RC, Miranda D, Amewu RK, Srivastava A, Gut J, Rosenthal PJ, O’Neill PM, Ward SA, Lopes F, Moreira R (2013). An endoperoxide-based hybrid approach to deliver falcipain inhibitors inside malaria parasites. ChemMedChem.

[185] Oliveira R, Miranda D, Magalhães J, Capela R, Perry MJ, O’Neill PM, Moreira R, Lopes F (2015). From hybrid compounds to targeted drug delivery in antimalarial therapy. Bioorg Med Chem.

[186] Witschel MC, Rottmann M, Schwab A, Leartsakulpanich U, Chitnumsub P, Seet M, Tonazzi S, Schwertz G, Stelzer Mietzner T, McNamara C, Thater F, Freymond C, Jaruwat A, Pinthong C, Riangrungroj P, Oufir M, Hamburger M, Mäser P, Sanz-Alonso LM, Charman S, Wittlin S, Yuthavong Y, Chaiyen P, Diederich F (2015). Inhibitors of plasmodial serine hydroxymethyltransferase (SHMT): cocrystal structures of pyrazolopyrans with potent blood- and liver-stage activities. J Med Chem.

